# Real-Time Estimation of *R*_*t*_ for Supporting Public-Health Policies Against COVID-19

**DOI:** 10.3389/fpubh.2020.556689

**Published:** 2020-12-22

**Authors:** Sebastián Contreras, H. Andrés Villavicencio, David Medina-Ortiz, Claudia P. Saavedra, Álvaro Olivera-Nappa

**Affiliations:** ^1^Centre for Biotechnology and Bioengineering, Universidad de Chile, Santiago, Chile; ^2^Departamento de Ingeniería Química, Biotecnología y Materiales, Facultad de Ciencias Físicas y Matemáticas, Universidad de Chile, Santiago, Chile; ^3^Division of Chemistry and Chemical Engineering, California Institute of Technology, Pasadena, CA, United States; ^4^Laboratorio de Microbiologia Molecular, Departamento de Ciencias de la Vida, Facultad de Ciencias de la Vida, Universidad Andres Bello, Santiago, Chile

**Keywords:** COVID-19, public-health policies, epidemiologic modeling, SARS-CoV-2, effective reproduction number *R*_*t*_

## Abstract

In the absence of a consensus protocol to slow down the spread of SARS-CoV-2, policymakers need real-time indicators to support decisions in public health matters. The Effective Reproduction Number (*R*_*t*_) represents the number of secondary infections generated per each case and can be dramatically modified by applying effective interventions. However, current methodologies to calculate *R*_*t*_ from data remain somewhat cumbersome, thus raising a barrier between its timely calculation and application by policymakers. In this work, we provide a simple mathematical formulation for obtaining the effective reproduction number in real-time using only and directly daily official case reports, obtained by modifying the equations describing the viral spread. We numerically explore the accuracy and limitations of the proposed methodology, which was demonstrated to provide accurate, timely, and intuitive results. We illustrate the use of our methodology to study the evolution of the pandemic in different iconic countries, and to assess the efficacy and promptness of different public health interventions.

## Introduction

**Figure 1:**
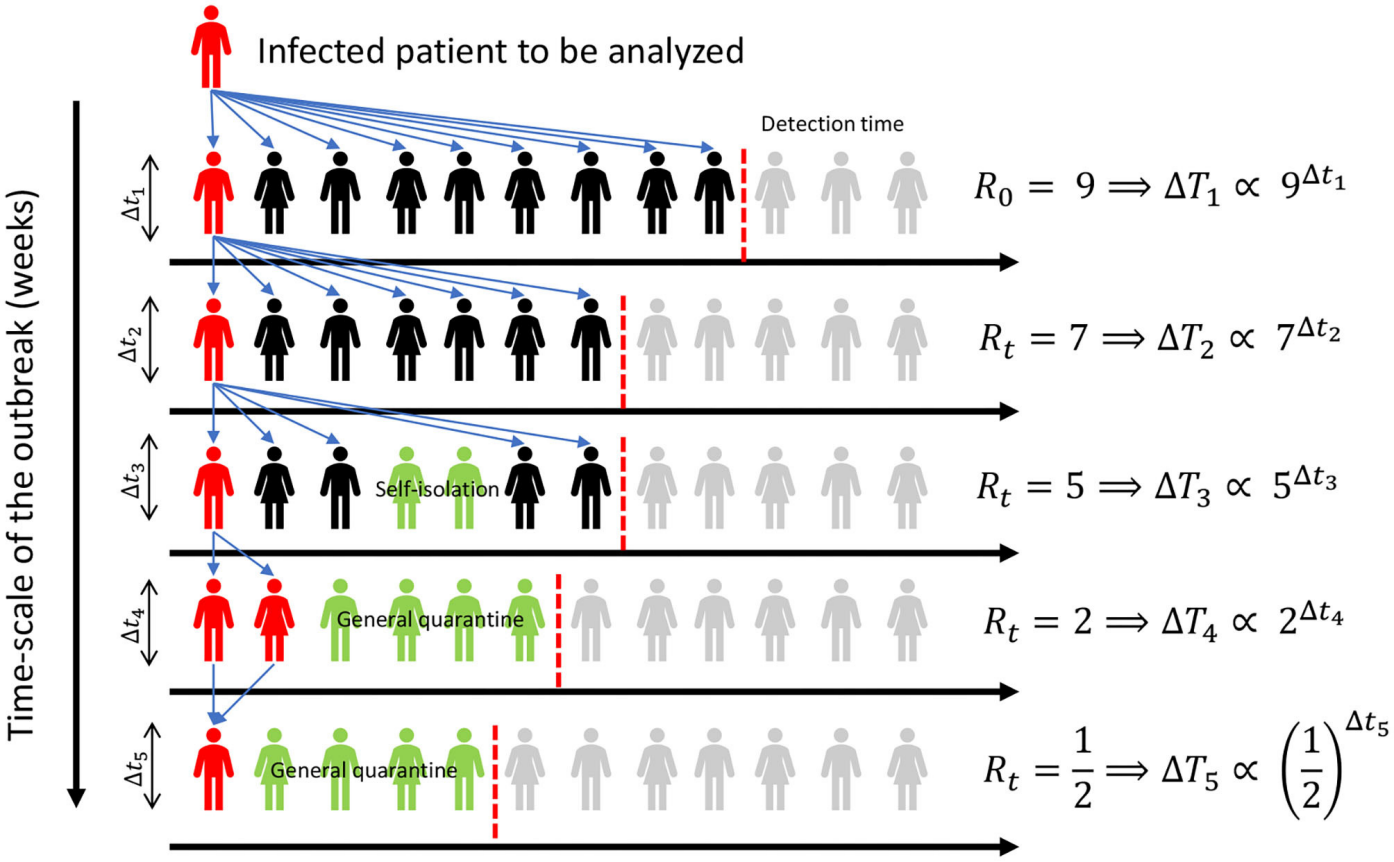
Different stages can be identified during the outbreak of an infection, characterized by the number of people that a single infected individual may infect. In the figure, a single infected individual (in red) can spread the virus among different individuals (in black), not reaching part of the population (in gray). Some individuals go into isolation (in green), effectively lowering their contagion chance. At the right-hand side of the plot, *R*_*t*_ represents the number of possible new infections caused by a single patient in each outbreak stage. In the first days of the outbreak, a single individual can infect several people before isolation, but as the amount of cases gets public awareness, health policies restricting movement and self-driven actions may help to control the outbreak, which is effectively captured by a decreasing *R*_*t*_.

Several mathematical models to fit public databases on the SARS-CoV-2 outbreak have recently been proposed (1–4). Despite their particularities, a great part share the same compartmental structure, inspired on the well-known SIR model (5). Besides the interest in modeling the spread of this virus, there is a need for indexes to evaluate the efforts made to prevent new cases, and to assess how likely a particular demographic group is to be infected. One of the parameters used for that means is the Basic Reproduction Number *R*_0_, which value represents the average number of persons a single infected individual infects if the population is fully susceptible and unaware of the virus (6). From its definition, *R*_0_ ≥ 1 indicates the outbreak has an exponential behavior, while *R*_0_ < 1 would account for a disappearing infection. When we study an ongoing outbreak, of which the population is aware and public measures are taking place, the parameter that represents the number of offspring infections produced by a single individual within a generation time [time between consecutive infections; (7)] is the Effective Reproduction Number *R*_*t*_ (8). An intuition on how it works is presented on Figure 1.

Even though several authors have claimed to have provided guidelines for obtaining both reproduction numbers (6, 9–11), the truth is that estimation methodologies remain somewhat cumbersome, especially for those readers untrained in mathematical modeling. The above raises a barrier to its applicability, as decision-making actors might consider it not simple enough to evaluate different public health plans. Regarding the mathematical approaches followed to estimate the different reproduction numbers, some rely on the population-level exponential growth rate (12), which, however, might work only in the early stages of the outbreak (13). Similarly, for later stages, compartmental mathematical models have been used to infer the virus's spreading rate and subsequently calculate the reproduction number (14–16). Chen et al. (1) uses a time-dependant SIR-model, where the different involved rates are allowed to change over time. After numerically finding the value that minimizes a curve-fitting functional, they evaluate the basic reproduction number considering two types of spreaders. Alternatively to these approaches, Luchini et al. (17) proposes a mathematical model that allows evaluating the convexity/concavity of trends in epidemiological surveillance data, evaluating the progression of the pandemic based on changes between acceleration and deceleration based on epidemiological metrics. The resulting plots of convexity or concavity, which would describe pandemic acceleration or deceleration, can be used as an adjunct criterion to the analysis of surveillance data to assess the pandemic's evolution (18).

In the present work, we propose a useful and simple methodology to calculate *R*_*t*_ directly from available epidemiological data in real time during an outbreak. The key feature of this practical methodology is that no specific knowledge in mathematics or scientific computation is needed to generate estimations of this parameter, thus being particularly handy for its use for day-to-day assessment in public health matters. We provide numerical insights on the accuracy of the proposed methodology, analyzing its stability considering different controlled levels of Gaussian noise. As our methodology does not involve a parameter fitting stage, which would be needed if solving the SIR system numerically to represent continuous trends, we can use it to evaluate the immediate impact of the different actions used to prevent the spread of SARS-CoV-2. In a case study, we assess the effect on *R*_*t*_ of the different ongoing measures taken by the Chilean government, and we compare their result with the current panorama of different iconic countries.

## Methods

Assuming the outbreak follows approximately an SIR model (Equations 1–3), we consider three compartments: susceptible *S*, healthy individuals, susceptible to be infected, infected *I*, individuals that have already contracted the virus, and removed *R*, individuals that are no longer infectious nor susceptible. Therefore, the dynamics are represented by:

(1) $$\begin{array}{l} S^{\prime }=-\frac{\beta \left(t\right)SI}{N}, \end{array}$$

(2) $$\begin{array}{l} I^{\prime }=\frac{\beta \left(t\right)SI}{N}-\gamma \left(t\right)I, \end{array}$$

(3) $$\begin{array}{l} R^{\prime }=\gamma \left(t\right)I. \end{array}$$

In particular, Equations (1) and (2) can be combined applying the chain rule and the derivative of the inverse function theorem, so we can write:

(4) $$\begin{array}{l} \frac{dI}{dS}=\frac{\frac{\beta \left(t\right)SI}{N}-\gamma \left(t\right)I}{\frac{-\beta \left(t\right)SI}{N}}\;\Leftrightarrow \;\frac{dI}{dS}=-1+\frac{\gamma \left(t\right)}{\beta \left(t\right)}\frac{N}{S}. \end{array}$$

In terms of the parameters of the SIR model, we can calculate *R*_*t*_ as the ratio between the time-dependant infection and recovery rates, β(*t*) and γ(*t*), respectively (1, 9), multiplied by the probability of finding a susceptible individual $\left(\frac{S}{N}\right)$:

(5) $$\begin{array}{l} R_{t}\left(t\right)=\frac{\beta \left(t\right)}{\gamma \left(t\right)}\frac{S}{N}. \end{array}$$

Note that, from its definition, not only variations in the infection and recovery rates affect the value of the effective reproduction number *R*_*t*_, but also the depletion of the susceptible pool. Using Equation (5), $\frac{\gamma \left(t\right)}{\beta \left(t\right)}\frac{N}{S}=\frac{1}{R_{t}\left(t\right)}$. Therefore, Equation (4) can be re-written as

(6) $$\begin{array}{l} \frac{dI}{dS}=-1+\frac{1}{R_{t}\left(t\right)}. \end{array}$$

Equation (6) can be discretized in an interval [*t*_*i*−1_, *t*_*i*_] where we can assume that *R*_*t*_(*t*) = *R*_*t*_(*t*_*i*_) is constant:

(7) $$\begin{array}{l} R_{t}\left(t_{i}\right)=\frac{1}{\frac{\Delta_{i}I}{\Delta_{i}S}+1}. \end{array}$$

Extending the classical SIR model to consider also deaths, a population balance dictates the discrete differences to follow Δ_*i*_*S* + Δ_*i*_*I* + Δ_*i*_*R* + Δ_*i*_*D* = 0. Then, Equation (7) takes its final form.

(8) $$\begin{array}{l} R_{t}\left(t_{i}\right)=\frac{1}{1-\frac{\Delta_{i}I}{\Delta_{i}I+\Delta_{i}R+\Delta_{i}D}}\Leftrightarrow R_{t}\left(t_{i}\right)=\frac{\Delta_{i}I}{\Delta_{i}R+\Delta_{i}D}+1. \end{array}$$

The last expression can be further simplified noting that Δ_*i*_*I* = (−Δ_*i*_*S*) − (Δ_*i*_*R* + Δ_*i*_*D*), namely, the variation on active cases Δ_*i*_*I* equals new infections (−Δ_*i*_*S*) minus the new -clinical- recoveries plus new deaths (Δ_*i*_*R* + Δ_*i*_*D*). Therefore, Equation (8) can also be expressed as:

(9) $$\begin{array}{l} R_{t}\left(t_{i}\right)=\frac{-\Delta_{i}S}{\Delta_{i}R+\Delta_{i}D}, \end{array}$$

or, in words,

(10) $$\begin{array}{l} R_{t}\left(t_{i}\right)=\frac{\text{New Infections}}{\text{New Recoveries }+\text{ New Deaths}} \end{array}$$

This last expression is particularly intuitive, it is straightforward to apply to understand the disease progression: when *R*_*t*_ > 1 there are more new infections than recoveries, thus the number of infecting individuals in the population is increasing, while for *R*_*t*_ < 1 the number of infecting individuals must be decreasing for the opposite reason.

###  Reach, Advantages, and Limitations of the Proposed Methodology

Equation (8) stands in front of other methods because of its simplicity and usability, as there is no need for specific mathematical or scientific computing knowledge for obtaining realistic values of *R*_*t*_ for a given population during an epidemic or pandemic outbreak. However, due to the nature of its dependence on real-time data, uncertainties on the input values would have a significant effect on the outcome. *A priori*, several practical uncertainty sources could affect the different variables that determine *R*_*t*_, mainly related to the non-uniformity in the case-reporting protocols, temporal delay on diagnosis, and other report or measurement biases (19). Since most common uncertainties are related to the time frame between contagion, sampling, detection and report (temporal misclassification), recovery criteria (PCR or fix quarantine time after which the patient is considered healed), we suggest to perform a data-pretreatment stage, where delayed curves could be corrected to the day of contagion before applying moving averages to smooth trends and estimate their variability while *R*_*t*_. The use of an one-week averaging window is suggested for minimizing the impact of the “weekend-effect” (20).

Another limitation of the proposed methodology relates to the low-case limit, where the different quantities involved in Equation (8) are (or are close to) zero. This situation may happen in the event of a sudden outbreak or a second wave in an already declared pandemic, where sensible sanitary measures in order to control the spread should be taken disregarding the value of any model-derived parameter. Moreover, in such cases, where contacts are known, and the number of cases remains low, contact-tracing is useful and should take place disregarding the calculated value of *R*_*t*_ (21). As this parameter relies on the hypotheses of the SIR model, it should be used with care; countries with strong heterogeneity between regions, the existence of different delays both in the infectious timeline and in the reporting procedure might affect its value (14, 19).

Estimations of the effective reproduction number with other methods do not necessarily match in value the ground-truth reproduction number of the disease *R*_*t*_ (22). Yet, they expose the same tipping point when they reach the value 1. This is the typically addressed tipping point which separates exponential growth from exponential extinction at the first stages of an outbreak. Nevertheless, other dynamics than exponential can arise from non-pharmaceutical interventions (NPIs), as the test-trace-and-isolate strategies (23), and in later stages of the outbreak.

## Results

### On the Accuracy of the Proposed Methodology

**Figure 2:**
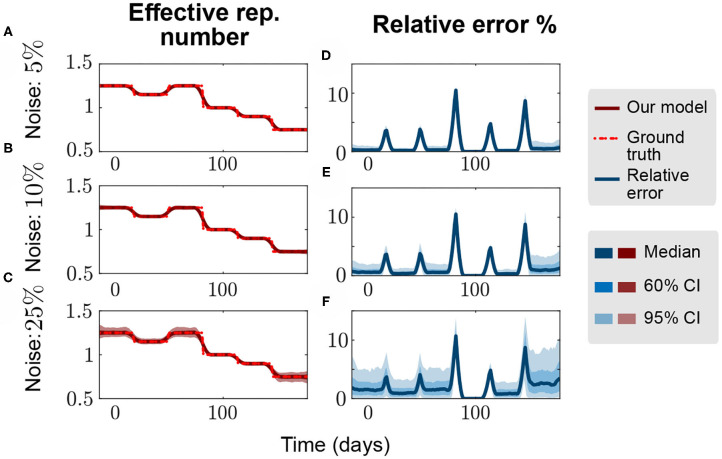
Numerical accuracy of the proposed methodology. We try to infer the values of *R*_*t*_ of a noisy time series generated from a SIR model with several change points spaced by 4 weeks (dotted red line–ground truth). **(A,D)**, **(B,E)**, and **(C,F)** represent the effective reproduction number and the relative error for 5%, 10%, and 25% proportional noise, respectively. The spreading rate β is such that, for a fixed recovery rate γ = 0.1 we obtain the ground-truth *R*_*t*_ = [1.25, 1.15, 1.25, 1.00, 0.90, 0.75] plotted. Initial conditions, *N* = 50 million people, *I*_0_ = 100, *S*_0_ = *N* − *I*_0_, and *R*_0_ = 0 individuals.

To evaluate the accuracy of the proposed methodology, we set up a numerical experiment to infer known values of *R*_*t*_ using a simulated SIR model with different levels of Gaussian noise and several *R*_*t*_ change points as a ground-truth. These change points may account for notable drifts in the population's behavior or for the application of different non-pharmaceutical interventions (NPIs). The standard methodology consisted of the use of 1-week rolling averages for the calculated *R*_*t*_, and we simulated different levels of noise to be added to the ground-truth Δ*I* and Δ*R* values. For a noise level *l* = {5%, 10%, 25%}, we defined noisy variables (Δ*I*)_noise_ = (Δ*I*)_model_(1 + *l*N(0, 1)), and we took random samples in a Monte Carlo-like experiment to build up confidence intervals (Figure 2, *n*_sample_ = 500). We can observe that, as the change points are discontinuous for $R_{t}^{\text{data}}$, the relative error is higher in the transitions. However, the method quickly captures the real *R*_*t*_ value for the different levels of noise in a period of less than the averaging-window time. This readily demonstrates the accuracy and robustness of the proposed methodology to capture different values of the time-dependent reproduction number *R*_*t*_ under the assumptions of this work.

### Effect of Non-pharmaceutical Interventions (NPIs) on the Spread of COVID-19

**Figure 3:**
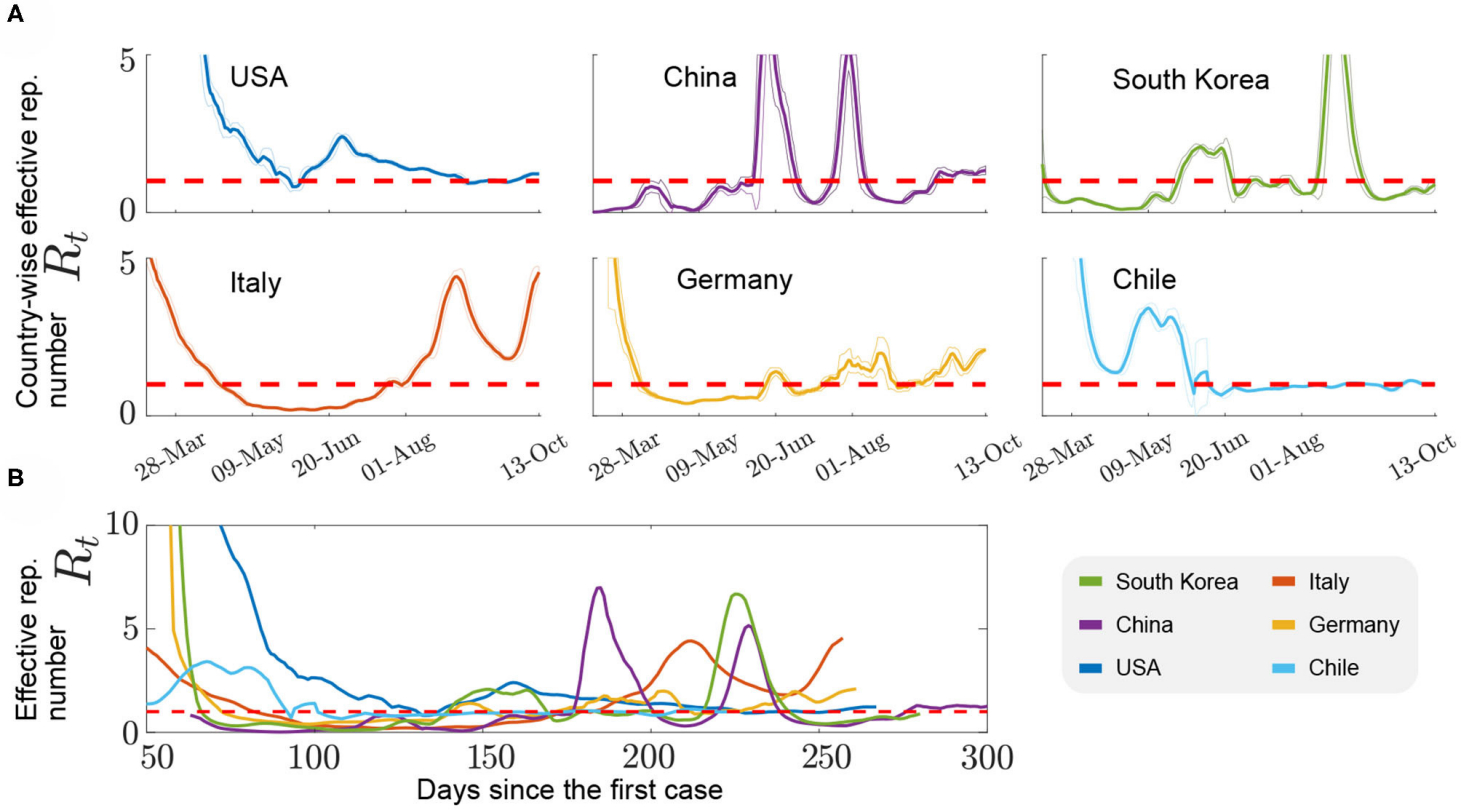
Comparative analysis of the values of *R*_*t*_ for different countries, using a moving average window of ± 3 days, in both daily reports and the calculated *R*_*t*_. The control threshold is represented by a horizontal red dashed line (*R*_*t*_ = 1). **(A)** Daily trends for *R*_*t*_ in different countries, from March 28 to October 13. **(B)** *R*_*t*_ trends from the day of detection of the first case, and their standard deviation in the moving average time-frame (confidence interval defined as mean plus/minus standard deviation). Note that the plot starts on day 50 after the first case report. Official data from Worldometers.info (October 13, 2020).

Countries that successfully control case numbers should exhibit *R*_*t*_ trends consistently lower than 1. A quick control of the viral spread can be recognized by an earlier decrease in their *R*_*t*_ trends when comparing them from the day of the first reported infection (Figure 3B). The efficacy of Non-Pharmaceutical Interventions can be assessed by the magnitude of the negative slope of the curves. Most countries also show second viral spread waves and even third ones. Noteworthy, those waves seem to be uniformly separated by a period of 2 months, even in cases like Germany, in which low amplitude waves took place with *R*_*t*_ slightly above the control threshold. South Korea seems again to be an exception, with second and third waves separated by approximately 3 months. There is also a notable difference in how second and third waves develop considering western and eastern countries, with the former appearing later than in China and Korea. Regarding peak amplitudes, it is noteworthy that in second and third waves, China, South Korea, and Italy (together with other non-shown European countries) show higher peaks than in other nations. However, this might also be a numerical effect since estimation methodologies for *R*_*t*_ do not agree well in the low case-number scenario. German and Chilean cases, among the countries shown in Figure 3A, seem to have been more successful in controlling the appearance of new spread waves, although using very different NPIs. However, Germany is lately following an ascending trend. In the case of Chile, the Government adopted a strict lockdown approach with border closure made effective 2 weeks after detection of the first case, compulsory quarantine with restriction of travel, movement, and meeting of people inside and between areas with higher case rates, restrictions in the commerce and closure of public and private schools, universities, restaurant, cinemas, shops, and public parks and other heavily transited meeting places, and compulsory use of face masks and hand disinfection measures in public places. Restrictions were applied differently by city, area, and region, depending on the number of cases, and changing over time. In Germany, governmental measures were radically different, and did not include severe lockdown measures or quarantines, relying only on people's compliance, high test rates, and high-surveillance and contact tracing policies.

Additionally, different sets of governmental measures were applied in Chile during the first months, sometimes with unfortunate public announcements that lead people to misinterpret the evolution of the pandemics and related measures, which are reflected by changes in the *R*_*t*_ number in time. For instance, in March 27, the Chilean Government declared a partial quarantine for districts with high case-density in Santiago (capital city concentrating 42% of the national population) and other cities, which were slowly applied to other districts. From May onward, contagion in Chile shows a steep increase that was maintained for a couple of weeks but then decreased to approach the control threshold, which can be related to new quarantine measures applied afterwards to more districts in Santiago and other cities. We can observe a somewhat irregular oscillatory behavior around *R*_*t*_ = 1, reflecting the efforts of the *step by step* governmental plan, which systematically release restrictions to boost economical activities, from July 19 on (24).

The USA case deserves special attention. Besides showing a comparatively slower and late decrease in *R*_*t*_ to control the first wave, which took more than three months to be lowered, revealing an inefficient approach to the control threshold, with only short periods around or below 1. Since the country's total population is high, values of *R*_*t*_ above one represent a catastrophic effect in terms of risk to the general population, with 51,564 new cases in October 13th (25).

## Discussion

We have developed a fast and simple methodology to estimate the Effective Reproduction Number *R*_*t*_ directly from raw real-time data of an evolving epidemic outbreak. Our results have also shown that this index can be a useful decision parameter to evaluate the impact of Non-Pharmaceutical interventions (NPIs) in controlling COVID-19. We studied the accuracy of the proposed methodology in a numerical experiment, concluding that results remain precise even when including a white noise of 25%. The simplicity of the proposed method to calculate *R*_*t*_ (Equation 8) remarks its applicability, and our analysis of *R*_*t*_ trends in different countries during the current SARS-CoV-2 outbreak highlights how it can be applied to assess both the speed of reaction and the efficacy of public-health measures. This provides decision-makers with a simple and easily calculable tool to timely understand the impact of their policies. As the proposed equation does not need vast volumes of data, it results particularly handy for its use when data resolution is not high enough to fit continuous models, in the analysis of short-time trends, or to compare different regions in the world or even inside a single country with different time density of data. This parameter should be calculated only for populations with shared characteristics, as it relies on the hypotheses of typical compartmental models (14). Countries with high heterogeneity in population density, climate, and behavior of different economic classes should be studied with care. The epidemiologic scenario of those zones concentrating the most significant part of the population would mask the spreading dynamics' local features. This case is especially highlighted in Chile, where the national average is dragged by the city of Santiago, not reflecting the situation of the most extreme regions, where the progress of the infection has been exponential even after remission in the capital, which in turn warns of the need to analyze local data in the same detail as national data. Consistently, as varied as the uses for the proposed methodology are the opportunities to improve it.

We look forward to seeing how this contribution of a real-time estimator of *R*_*t*_ would impact how we analyze the ongoing contingency and how the scientific and decision-making community would adapt it to tailor propagation models and obtain better and timely insights on the application of emergency public-health policies.

## Data Availability Statement

Publicly available datasets were analyzed in this study. This data can be found at: https://www.worldometers.info/coronavirus/; https://www.gob.cl/coronavirus/cifrasoficiales/.

## Author Contributions

SC, DM-O, and HV: conceptualization, methodology, and investigation. ÁO-N, SC, and CS: validation. SC, DM-O, HV, and ÁO-N: writing, review, and editing. ÁO-N and CS: supervision and project administration. ÁO-N: funding resources. All authors contributed to the article and approved the submitted version.

## Conflict of Interest

The authors declare that the research was conducted in the absence of any commercial or financial relationships that could be construed as a potential conflict of interest.
